# Supplementary material for: Transcriptome Analysis Reveals Vimentin-Induced Disruption of Cell–Cell Associations Augments Breast Cancer Cell Migration
Source: Cells. 2022 Dec 13;11(24):4035. doi: 10.3390/cells11244035 (PMC9776984; doi:10.3390/cells11244035)
Supplement: Supplementary file 1 [file cells-11-04035-s001.zip › DEGs and enrichment analysis.zip!/DEGs and enrichment analysis/WT_VIMvsVector KEGG/DOWN/WT_VIMvsVector_DOWN.pathway_map.zip!/WT_VIMvsVector_DOWN.pathway_map/src/hsa04217.html]

hsa04217


- hsa:330

- Down regulated genes

ENSG00000023445(-2.12138419902495)

- hsa:6775

- Down regulated genes

ENSG00000138378(-3.50780456717299)

- hsa:824

- Down regulated genes

ENSG00000162909(-2.04343414211511)

- hsa:824

- Down regulated genes

ENSG00000162909(-2.04343414211511)

Close
